# The Glial Coupling in the Developing Brain: Astrocytes, Oligodendrocytes and Myelin Formation

**DOI:** 10.3390/metabo16100774

**Published:** 2026-10-13

**Authors:** Maria Ester Canepa, Chiara Santucci, Daiana Mariano, Sara Mangini, Andrea Calandrino, Luigi Gagliardi, Andrea Petretto, Pasquale Striano, Luca Antonio Ramenghi

**Affiliations:** 1 Department of Neuroscience, Rehabilitation, Ophthalmology, Genetics and Morther-Child Health, University of Genoa, 16132 Genoa, Italy; mestercanepa@gmail.com (M.E.C.); chiara.santucci@yahoo.it (C.S.); daiana.mariano@edu.unige.it (D.M.);; 2 Neonatal Intensive Care Unit, IRCCS Istituto Giannina Gaslini, 16132 Genoa, Italy; saramangini@gaslini.org (S.M.); l.gagliardi@neonatalnet.org (L.G.); 3 Core Facilities—Clinical Proteomics and Metabolomics Unit, IRCCS Istituto Giannina Gaslini, 16132 Genoa, Italy; 4 Pediatric Neurology and Muscular Disease Unit, IRCCS Istituto Giannina Gaslini, 16132 Genoa, Italy

**Keywords:** glia, brain, neonate, elderly, development

## Abstract

Astrocytes and oligodendrocytes contribute to the metabolic, homeostatic and structural organization of the developing brain, yet their interactions evolve across development and remain incompletely characterized during the earliest stages of life. This narrative review examines astrocyte–oligodendrocyte coupling in relation to myelin development and addresses the question of which cellular and metabolic mechanisms support oligodendrocyte maturation and white-matter metabolism before mature astrocytic functions are fully established. Evidence from preterm and ill term neonates is considered together with experimental and adult or elderly studies used exclusively for secondary mechanistic comparison. Astrocytes and oligodendrocytes participate in interconnected metabolic, developmental and homeostatic processes, while perinatal white-matter injury may combine reactive astrogliosis, pre-oligodendrocyte injury and maturation arrest. Neuroimaging, including quantitative susceptibility mapping, synthetic MRI, cranial ultrasound and molecular or myelin-sensitive imaging, provides complementary but indirect information on the structural and maturational consequences of these processes, whereas omics approaches may characterize their molecular environment. Radiomics similarly provides indirect tissue-level biomarkers rather than cell-specific measures of glial biology. Dysglycaemia further illustrates the dependence of developing white matter on coordinated cellular energy handling and the potential relevance of immature astrocyte–oligodendrocyte interactions during metabolic instability. Evidence from very early development suggests that oligodendrocyte maturation occurs within a changing cellular environment in which astroglial maturation, neuronal and vascular processes, and immune and metabolic mechanisms coexist. The Crossword and Foil Effects are proposed as hypothesis-generating frameworks to organize these intersecting developmental, metabolic, cellular, imaging and molecular dimensions rather than as experimentally validated causal models. Overall, no single cellular substitute for mature astrocytic functions is identified; instead, oligodendrocyte maturation appears to emerge within a developmentally changing division of metabolic, homeostatic and signaling labour across astroglial, neuronal, vascular and immune compartments.

## 1. Introduction

Glia are described as the architects of the neural extracellular matrix [1]. Since they collaboratively shape circuit development through the instruction, stabilisation, and elimination of wiring programs, “a disruption of this coordinated logic, beyond dysfunction of any single glial or neuronal process, underlies vulnerability to neurodevelopmental and neuropsychiatric disease” [2]. Among glia, astrocytes and oligodendrocytes are key cells distributed in the blood–brain barrier (BBB) and in the neurovascular unit (NVU). Astrocytes (ASTs) and oligodendroglia (OLG) actively interact in myelin development and related brain diseases, as shown in adult studies [3], considering how AST-OLG interactions are involved in protecting the integrity of the blood–brain barrier (BBB) and regulating synaptogenesis and neural activity. Recent studies further suggest that AST-OLG interactions are associated with myelin-related diseases, such as multiple sclerosis and Alzheimer’s Disease [4,5,6]. Studying these cells in the neonatal brain, a systems biology approach may be particularly relevant, as suggested by De Luca et al. [7]. In this context, neurons, glia, the extracellular matrix and the NVU contribute to the complexity of synaptic plasticity, which is important for learning and cognitive functions [8].

**Figure 1:**
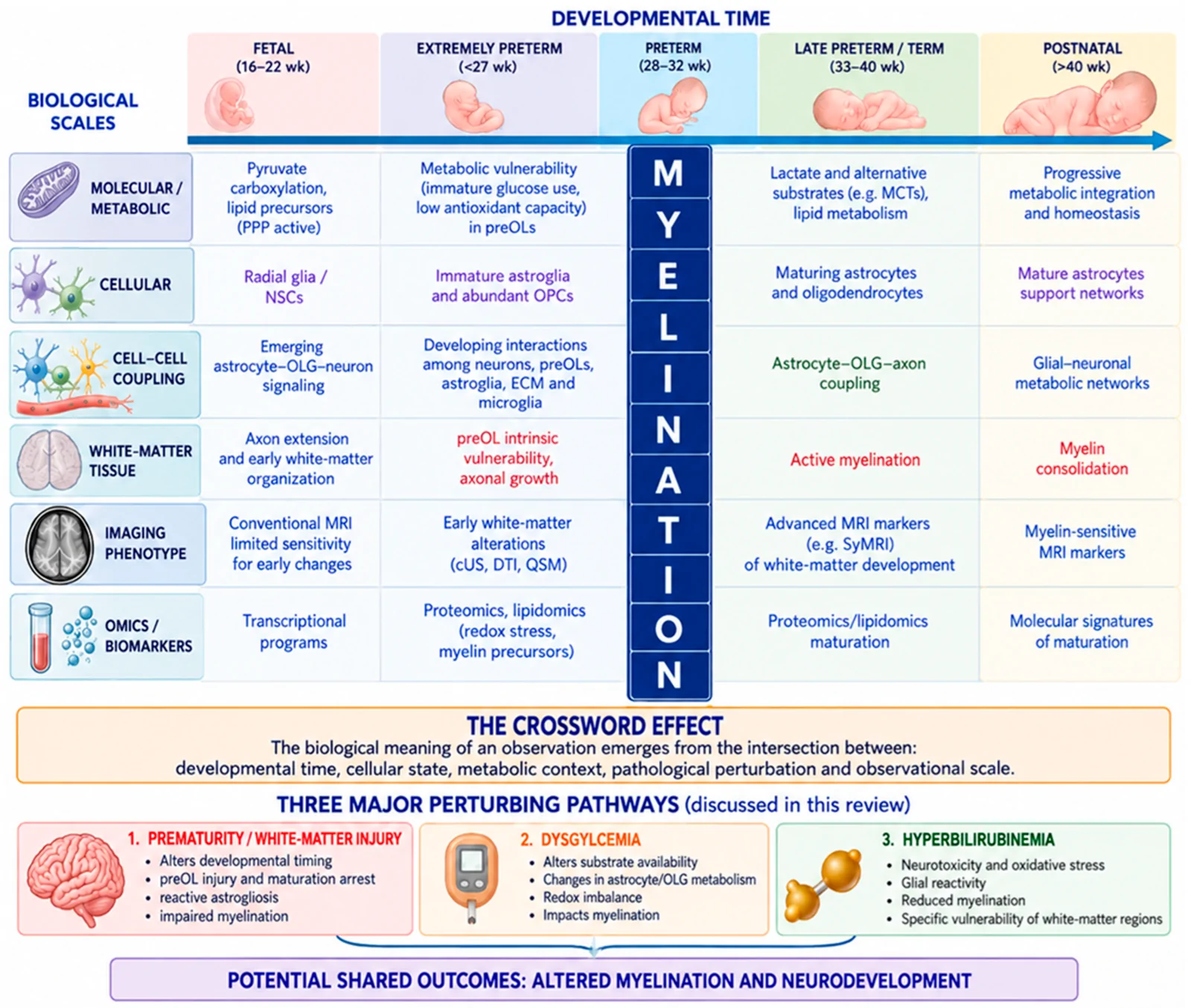
The Crossword Effect of the multidimensional determinants of myelination in the developing brain. The Crossword Effect is presented as a hypothesis-generating conceptual framework integrating the developmental, metabolic, cellular, imaging and molecular dimensions discussed in this review. It is intended to organize their potential intersections rather than to represent an experimentally validated mechanistic or causal model.

In a previous work, we discussed neurovascular unit coupling in neonatal health and disease in comparison with findings from the literature on neurodegenerative disorders in older populations, considering astrocytes and oligodendrocytes as two among the essential pieces of this pathophysiological and cellular puzzle called “preterm brain injury” [9]. Kugler et al. described NVU features through the neuro-glia-vascular unit framework [10]. It consists of multiple cell types, and its functionality goes beyond the resulting neurovascular coupling, with cross-component links of signaling, metabolism, and homeostasis. In neonates, established astroglial markers GFAP, S100 and AQP4 are neither sensitive nor specific enough to embrace the entire population of astrocytic/astroglial cells in the developing human brain at midgestation and cannot distinguish between different maturation steps [11]. Conversely, developmental age, region and, if possible, astroglial subtype should be considered when choosing appropriate astroglial markers. According to the study by Holst et al., AQP4 characterizes a brain-wide water transport system in cerebral cortex at midgestation and it may characterize radial glial cells destined for the astroglial lineage [11]. YKL-40 and C3d, previously found in reactive astrocytes, stain different subpopulations of astrocytes/astroglial progenitors in developing hippocampus at midgestation and may characterize specific groups of ‘developmental astrocytes’ [11]. Conceptually informed by the Crossword Effect illustrated in Figure 1 and applied elsewhere to integrate context-dependent biophysics, biochemistry, pharmacology, and anatomy within pathophysiological dynamics [12], the present narrative review will explore the biochemical profile of myelin in interaction with glia cells in the developing brain health and disease. Thereafter, omics pipelines based on blood and other biological sample types are described to identify biomarkers potentially supporting the diagnosis of brain injury in preterm neonates.

**Research questions**: Who supports oligodendrocyte maturation and white-matter metabolism before mature astrocytic functions are fully established?

**Terminology choice, search strategy and narrative review reporting**: Articles were included irrespective of publication type (except for retracted studies) from inception to 10 September 2026. No language restriction was applied. The search strategy comprised keywords related to “astrocyte”, “oligodendrocyte”, “preoligodendrocyte”, “myelin” in PubMed, CINAHL and Embase. Eligibility was defined according to the population, biological exposure and outcomes relevant to the review question. Studies addressing preterm neonates and ill term neonates were considered when they investigated glial cells and processes potentially related to glial uncoupling, with particular attention to astrocyte–oligodendrocyte interactions. Healthy term newborns were considered as the comparator population, when applicable. Outcomes of interest were neonatal brain injury and alteration of developmental myelination. Given the narrative and mechanistic scope of the review, experimental, elderly and adult studies were additionally considered when they provided biological evidence relevant to the interpretation of glial coupling or uncoupling mechanisms in the developing brain. Evidence derived from experimental models and adult or elderly populations was used exclusively for secondary mechanistic comparison and was not considered equivalent to, or directly generalizable to, evidence from the developing human brain. The reporting of this narrative review was informed by the SANRA checklist for reporting narrative reviews [13]. Throughout this review, demyelination, myelin loss, delayed or impaired myelination, and myelination failure are used according to the specific biological process or outcome described in the cited studies and should not be interpreted as interchangeable terms, particularly in the developing brain.

## 2. Astrocytes and Oligodendrocytes Across Development: A Temporally Evolving Metabolic Partnership

Given their involvement in maintaining BBB functions from molecular and physiological perspectives [14,15], astrocytes are extremely relevant in neonates and adults, especially after birth [16]. Accordingly, describing its functions separately could be complex rather than with a ramified approach on intertwining axes: metabolism (hypothalamic astrocytes) and myelination [17,18,19,20] directly considering anatomical localization and physiology function of BBB and NVU. and mechanisms and potential functions of neural cell competition (NCC), ranging from neural progenitors, neurons, astrocytes and oligodendrocytes to microglia. Physiological NCC governs cellular survival, proliferation, arborization, organization, function and territorial colonization, whereas dysregulated NCC may cause neurodevelopmental disorders, accelerate aging and exacerbate neurodegenerative diseases [21], Primarily, astrocytes have been known as a metabolic support supplying neurons for decades [20]. With neurons, they build specific neural metabolic networks [22] to engage metabolic functions.

While neurons are involved in mitochondrial ATP generation, pentose phosphate pathway and neurotransmitter synthesis, astrocytes are associated with antioxidant capacity, precursor synthesis, lipid synthesis, glycogen synthesis, and glycolytic activity. Therefore, astrocytes work in tandem with oligodendrocytes for glucose and myelin metabolism as well as protective brain insult recovery [3,23,24,25,26,27]. Conversely, oligodendrocytes are not subordinate to astrocytes. Rather, they are reciprocally synchronized with astrocytes from biochemical and biophysical perspectives, depending on oligodendrocyte cell function. First, the glycolytic oligodendrocytes maintain myelin and axonal integrity [28] whose physiological coupling is mediated by K+ extracellular channels [29]. Second, variations in oligodendrocyte function may have immunological relevance to neurological health [30]. Oligodendrocytes may participate in local immune regulation in addition to their canonical roles in myelination and metabolic support. Under inflammatory conditions, their interactions with astrocytes, microglia and other immune cells may modify the cellular environment in which oligodendrocyte maturation and myelination occur. This immune dimension is particularly relevant to the developing brain, where inflammatory and haemodynamic disturbances may coexist with white-matter vulnerability [31].

Third, astrocytic and oligodendrocytic lineages share developmental regulatory mechanisms. In postnatal subventricular zone progenitors, the transcription factor Olig2 has been shown to promote both astrocytic and oligodendrocytic fates while suppressing neuronal differentiation [32]. This shared developmental regulation further supports considering astrocytes and oligodendrocytes as components of an evolving glial partnership rather than as independent cellular systems. Furthermore, nonsynaptic junctions on myelinating glia promote preferential myelination of electrically active axons [33].

Disruption of astrocyte–oligodendrocyte interactions in neonatal brain pathophysiology could result in functional asynchronization associated with myelin loss [34,35] as astrocyte-derived factors and oligodendrocytes regulate myelination [36,37] through the specific cells reported in Table 1. Moreover, cortical maturation is asynchronous in preterm newborns [38] similarly to the “Desynchronise, disconnect, reorganise, collapse theoretical framework” linking white matter pathology and cognitive dysfunction in vascular disease in older individuals with Alzheimer’s Disease [39]. Perinatal white-matter injury (WMI) provides a relevant example of disrupted astrocyte–oligodendrocyte interactions. Viaene distinguishes focal white-matter necrosis from diffuse white-matter gliosis, while emphasizing that these patterns may represent a pathological spectrum rather than entirely separate entities [40]. Importantly for the present review, diffuse WMI combines reactive astrogliosis with pre-oligodendrocyte injury and subsequent maturation arrest, thereby linking alterations in the astroglial environment to delayed developmental myelination. Axonal injury may accompany both focal and diffuse WMI, including diffuse lesions without overt necrosis [40].

Astrocytes are guardians of brain homeostasis across the lifespan [41] owing to balanced bidirectional crosstalk between the BBB and neural cells, as well as between astrocytes and oligodendrocytes in the central nervous system [2,3,14,15]. In pathophysiological conditions, alterations in astrocytic function may contribute to the broader cellular and homeostatic imbalance associated with perinatal brain injury [26].

Imaging studies may help characterise the demyelination in the context of neurodegeneration and microglia-mediated inflammation [41,42,43], particularly because microglia and astrocytes are important players in producing myelin in health and disease [44]. In neonates, illustrative examples are provided by the Gaslini Children’s Hospital group by Tortora et al. [45] and the Oliveri et al.’s group [46] who used quantitative susceptibility maps, an approach previously applied to the study of myelin in healthy newborns and in the paediatric imaging literature [47,48]. These studies considered two representative conditions potentially leading to brain injury in preterm and term infants, respectively: the intraventricular haemorrhage [45] (IVH) and the perinatal asphyxia [46]. In preterm neonates with normal brain MRI, all white and subcortical gray matter regions had negative magnetic susceptibility values (diamagnetic). Neonates with severe GMH-IVH showed higher positive magnetic susceptibility values (i.e., paramagnetic) in the centrum semiovale (0.0019 versus −0.0014 ppm; *p* < 0.001), temporal WM (0.0011 versus −0.0012 ppm; *p* = 0.037), and parietal WM (0.0005 versus −0.0001 ppm; *p* = 0.002) compared with controls. No differences in magnetic susceptibility were observed between neonates with mild GMH-IVH and controls (*p* = 0.236). Paramagnetic susceptibility changes occur in several normal-appearing WM regions of neonates with severe GMH-IVH, likely related to the accumulation of hemosiderin/ferritin iron secondary to diffusion of extracellular hemoglobin from the ventricle into the periventricular WM. Indeed, disrupted iron trafficking and ferroptosis are linked to perinatal brain injuries [49,50,51] as iron homeostasis could be regulated by extracellular vesicles [52,53]. Furthermore, iron homeostasis disruption could be associated with oxidative stress dynamics [53] which are involved in further brain lesions in preterm infants [54]. By analysing 74 MRI examinations performed during the first month of life, Oliveri et al. showed that, among neonates with neonatal encephalopathy who were treated with hypothermia and developed brain injury, birth asphyxia was associated with impaired myelination in the regions that are myelinated at birth or soon after birth (the posterior limbs of internal capsule, the thalami, and the lentiform nuclei), in the regions where the myelination process begins only after the perinatal period (optic radiations), and in the regions where this process does not occur until months after birth (anterior/posterior white matter).

Neuroimaging cannot directly resolve astrocyte–oligodendrocyte coupling in vivo; however, it provides complementary information on the structural and maturational consequences occurring within the developing white matter. These imaging approaches should therefore be considered complementary rather than interchangeable. QSM primarily reflects magnetic susceptibility sources, including iron-related tissue changes, whereas SyMRI derives quantitative tissue properties that may characterize maturational alterations. Cranial ultrasound provides bedside structural and maturational information but does not directly quantify myelin, while PET and MRI-based myelin imaging interrogate distinct molecular or tissue properties with modality-specific validation constraints. Several authors have addressed the challenge of visualising myelin through imaging techniques like PET and MRI [55]. Although Schmidbauer et al. reported that SyMRI software can detect delayed myelination in preterm neonates by generating different MR contrasts characterising tissue properties from a single acquisition of a multi-dynamic multi-echo (MDME)-FLAIR sequence [56], van der Weijden et al. [55] highlighted the scarcity of validation studies without reporting of the effects of tissue orientation, iron, B1 inhomogeneity and changes in water content on SyMRI. Since oligodendrocytes are involved in maturational brain processes, other authors have developed specific brain ultrasound imaging scores to evaluate cortical and white matter maturation in preterm infants and to predict neurodevelopment [57,58,59], as well as other conditions [60]. Nonetheless, brain ultrasound scoring systems remain challenging and have not yet been validated at a wider scale; moreover, no consensus version exists despite the 2021 Canadian attempt by Mohammad et al. to propose a standardized consensus score for classifying preterm brain injury [61].

## 3. Myelin Through Omics Eyes. A Further Possible Complement with Imaging Biomarkers?

Whereas neuroimaging primarily characterizes the tissue phenotype of altered white-matter development, omics approaches offer a complementary means of interrogating the molecular environment in which astrocyte–oligodendrocyte maturation occurs. Indeed, omics pipelines have further enhanced the research on myelin [62,63], mapping lipidome and proteome of myelin membranes in 2013 [62]. Proteomics and lipidomic analyses reveal dysregulated pathways associated with loss of sacsin in the autosomal recessive spastic ataxia of Charlevoix-Saguenay (ARSACS) [64]. Experimental analyses from primary astrocyte-enriched cultures derived from the cerebral cortex of neonatal rats further support the relevance of sacsin to astroglial biology [65]. Sacsin deletion induces aggregation of glial intermediate filaments [65]. At present, these molecular signatures cannot be considered cell-specific readouts of astrocyte–oligodendrocyte dysfunction. Their value may instead lie in defining metabolic, inflammatory, and vascular contexts that can subsequently be integrated with developmental and imaging phenotype.

Radiomics extends conventional neuroimaging by extracting quantitative features that may capture tissue heterogeneity beyond visual assessment. These features, however, remain indirect imaging biomarkers and should not be interpreted as direct measures of astrocytic or oligodendroglial biology. In neonatology, radiomics studies have investigated MRI-derived biomarkers and physiological parameters in at-risk neonatal populations, including neonates with hyperbilirubinemia (NH). Hyperbilirubinemia may impair neurodevelopment, requiring continued follow-up [66,67], and has also been associated with reduced myelination and increased glial reactivity [68]. Furthermore, a study on a preterm neonate with kernicterus [35] shows a further combined cerebellar axon/myelin loss, angiogenic sprouting, and neuronal increase in Vascular Endothelial Growth Factor (VEGF). These concomitant neural, myelin and vascular changes may provide an illustrative example of how disturbances across glial and neurovascular compartments can coexist in neonatal brain injury. In neonatal hyperbilirubinemia, radiomics studies have explored whether quantitative features extracted from T1-weighted MRI can distinguish acute bilirubin encephalopathy from normal myelination or unaffected controls. Liu et al. developed a machine-learning model based on radiomic features extracted from the basal ganglia and globus pallidus, reporting discriminatory performance between neonates with acute bilirubin encephalopathy and controls [69]. Similarly, Sun et al. developed radiomics-based models to distinguish acute bilirubin encephalopathy and neonatal hyperbilirubinemia from control groups [70]. Accordingly, the potential relevance of radiomics to astrocyte–oligodendrocyte biology lies in its ability to characterize the tissue phenotype with indirect biomarkers associated with altered white-matter development, rather than to identify the underlying cellular mechanism itself. Characterization of tissue phenotypes and glial cell activation is particularly sensitive to spatiotemporal variation in both neonates and older patients with Alzheimer’s disease [71,72,73,74,75,76].

## 4. Is Astrocyte–Oligodendrocyte Metabolism or Multisystem Brain Metabolism More Relevant to the Pathogenesis of Neonatal Brain Injury Associated with Dysglycaemia?

Crucially, astrocytes interact with neurons in the regulation of glucose metabolism [77] and may also regulate the survival of newborn hippocampal neurons in the adult brain [78], further highlighting the biochemical complexity of brain metabolism [79]. Neonatal cerebral glucose metabolism differs substantially from that of the adult brain. Studies using ^13C-labelled acetate indicate that astrocyte–neuron metabolic exchange is already active during this developmental period, although it is not yet organized as in the mature brain [77]. Glutamine transfer from astrocytes to neurons appears relatively efficient, whereas the reciprocal supply of neuronal glutamate is more limited [77]. The neonatal brain may compensate for this imbalance through greater anaplerotic pyruvate carboxylation. Glucose utilization also differs from the adult pattern, with a comparatively greater contribution of the pentose phosphate pathway relative to glycolysis and pyruvate dehydrogenase activity [77].

Within the scope of this review, the relevance of dysglycemia therefore lies not only in its association with neonatal brain vulnerability, but also in the dependence of the developing white matter on coordinated cellular energy handling. Metabolic instability may consequently represent one of the contexts in which immature astrocyte–oligodendrocyte interactions become particularly relevant.

Indeed, De Angelis et al. previously reviewed the literature on the role of hypoglycaemia as a risk factor for neonatal brain injury [80] and subsequent publications from Gaslini Children’s Hospital have focused on the potential role of continuous glucose monitoring as a neuroprotective strategy for preterm infants in the NICU [81,82,83]. Although hypoglycaemia thresholds are heterogeneous across studies and guidelines, the double-blind, randomized Sugar Babies Trial, published in *The Lancet*, showed that dextrose gel should be considered a first-line treatment to manage hypoglycaemia in late preterm and term babies during the first 48 h after birth [84], as further supported by Cochrane’s reviews [85].

## 5. Astrocytes and Oligodendrocytes Studies in Preterm Babies Born Before 27 Weeks of Gestation

What about neonates born before 27 weeks of gestation? The discussion should therefore move towards prenatal development. In the 2020 article “Astrocytogenesis: where, when, and how”, Akdemir et al. [86] describe how neural stem cells (NSCs), or radial glia, generate astrocytes through complex intrinsic and extrinsic cellular processes. This sequential series of events results in mature astrocytes that actively participate in CNS physiology. Conserved mechanisms regulate gliogenesis in the spinal cord and brain, although the process begins early, at embryonic day 11.5 (E11.5), in the spinal cord and later, at E18, in the brain. Whereas spinal cord astrocytes are derived from the ventricular zone (VZ), forebrain astrocytes originate from the ventricular–subventricular zone (V-SVZ). Furthermore, Vivi and Di Benedetto described astrocyte development across the lifespan by comparing mice and humans [16]. Specifically, they showed that at 20 weeks of gestation in humans (corresponding approximately to E16 in mice):

(a) Astrocytes begin to proliferate, mature, and differentiate after birth.
(b) The axons and dendrites begin to develop together with synaptic pruning.

Particularly, astrocytes and microglial cells both regulate the refinement of synaptic neuronal networks through the elimination of weak synapses during early postnatal developmental stages. Signaling molecules called “eat me” signals, such as C1q and phosphatidylserine (PS), recruited during these events have been identified, as well as binding partners located on the respective cells, like MEGF10 receptor on astrocytes and the receptors of the complement cascade on microglia cells. It remains unclear whether PS and C1q are directly implicated in the astrocyte-mediated synaptic elimination. Indeed, Roessmann and Gambetti [87] underscored the diffuse proliferation as the most significant finding in the evaluation of the perinatal damage, in both the acute state and in the long-term survivors in 1986. Specifically, they analysed the astrocytic reaction to various types of pre- and perinatal damage in the brain with the immunohistochemical method for glial fibrillary acidic protein. They found that the reactive gliosis could be detected as early as 20 weeks’ gestation. Moreover, reactive proliferation of the astrocytes could be seen already at 4 days after the insult. In addition to reacting to focal lesions, astrocytes also proliferated diffusely throughout the white matter.

**Figure 2:**
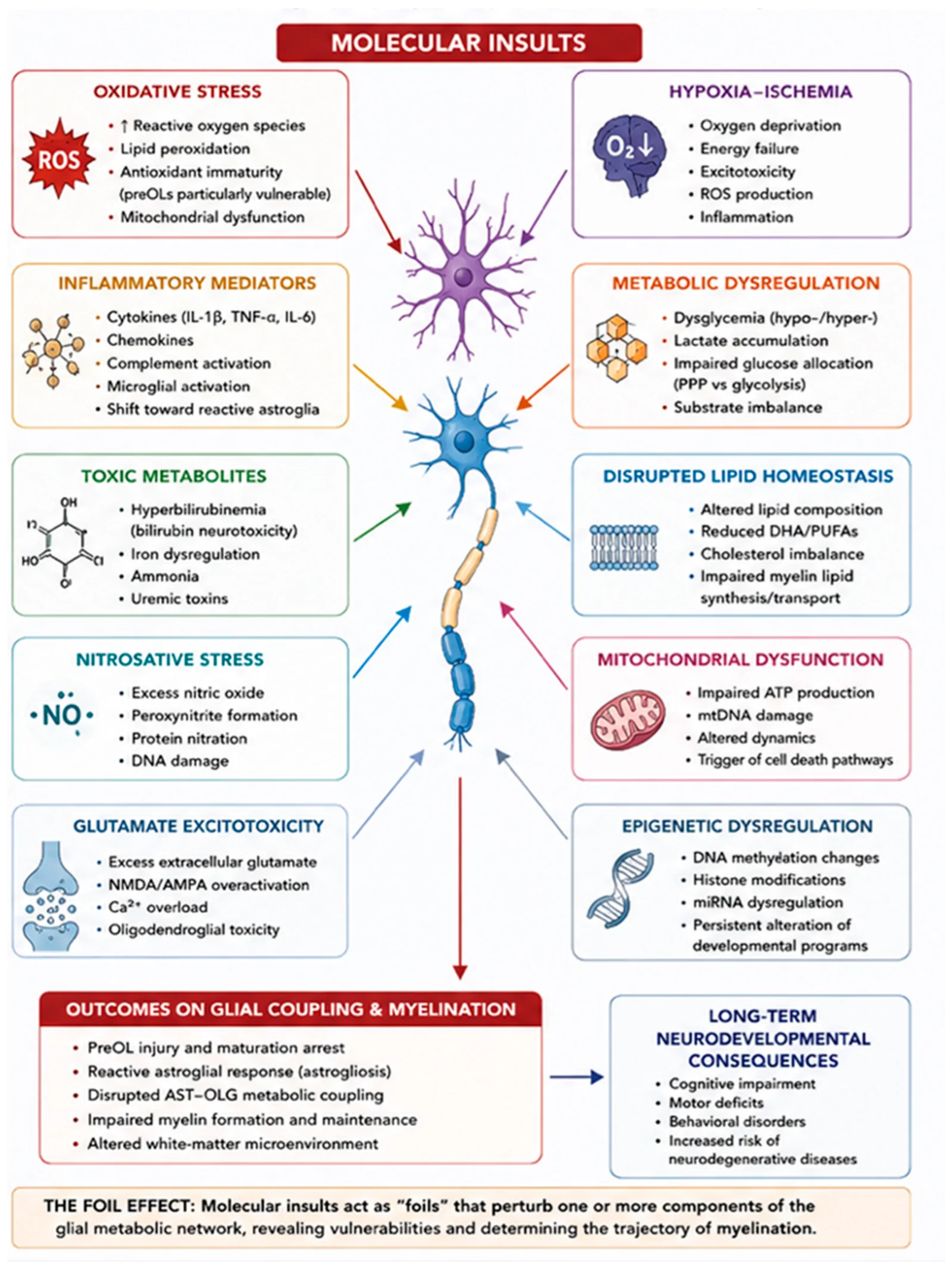
The Foil Effect. Molecular insults to glial coupling and myelination in the developing brain. The Foil Effect is presented as a hypothesis-generating conceptual framework illustrating how multiple molecular perturbations discussed in this review may intersect across the developing brain. It represents the authors’ conceptual interpretation and should not be interpreted as an experimentally validated causal sequence.

Since astrocytes are the first cells to react to a brain insult, the state of astrocyte reactivity is considered one of the key factors discriminating between either the initiation of endogenous reparative mechanisms compensating for aberrations in the structures and functions of nervous tissue or the triggering of neurodegeneration. Reviewing the current literature on this topic, Gargas et al. [88] illustrate that the responses of activated cells are modulated by neighboring neural cells, which exhibit broad immunomodulatory and pro-regenerative properties by secreting a plethora of active compounds (including interleukins and chemokines, neurotrophins, reactive oxygen species, nitric oxide synthase and complement components), which are engaged in cell crosstalk in a paracrine manner. As the developing nervous system is extremely sensitive to the influence of signaling molecules, even subtle changes in the composition or concentration of the cellular secretome can have significant effects on the developing neonatal brain. This phenomenon is analogous to the Foil Effect in Figure 2, which we have conceptualised elsewhere as a hypothesis-generating framework in which multiple subtle molecular insults could potentially contribute to primary and secondary brain injury [9]. Taken together, these observations span different biological scales—from cellular maturation and metabolic support to tissue-level imaging phenotypes and systemic molecular signatures. We therefore use the Crossword and Foil Effects as hypothesis-generating frameworks to organise, rather than replace, the established evidence across these levels.

Oligodendrocytes are equally important for disentangling astrocyte–oligodendrocyte interactions in brain injury before 27 weeks of gestation. During this developmental period, oligodendrocyte precursors are susceptible to hypoxia and ischaemia in the premature mouse brain, as myelination failure in chronic white matter lesions is related to a combination of delayed preOL degeneration and preOL maturation arrest [89]. Notably, the oligodendrocyte precursors die 24 h after induced hypoxia, whereas astrocytes survive [90]. The persistence of a susceptible population of preOLs renders chronic white matter lesions markedly more vulnerable to recurrent hypoxia–ischemia [89]. For comparison, studies in adult mice have shown that white-matter stroke induces a unique oligo-astrocyte niche that inhibits recovery [91]. In human neonatal autopsy samples [92], the primary mechanism of myelination failure involves a disrupted cellular response whereby preOLs fail to differentiate in diffuse astrogliotic lesions. PreOL maturation arrest converts chronic WMI to a more immature state related to the burden of astrogliosis. In humans, microglia mediate neuroinflammation and neuroplasticity after stroke [43].

**Conclusions**. No single cellular substitute may exist. Rather, oligodendrocyte maturation appears to emerge within a developmentally changing division of metabolic, homeostatic and signaling labour across astroglial, neuronal, vascular and immune compartments. Perinatal injury may disrupt this evolving division of labour while simultaneously arresting oligodendroglial maturation, thereby altering subsequent myelination. As previously highlighted in the literature [93], myelination, neuronal migration, and synaptogenesis should be considered when informing targeted interventions aimed at fostering neurodevelopmental outcomes in preterm infants.

## Figures and Tables

**Table 1:** Key cells in myelination per localization.

Cell Name	Myelin Production Hub	Special Aspects
Olygodendrocytes	Central Nervous System (spinal medulla; encephalus)	It can myelinate multiple portions of axons simultaneously
Schwann cells	Periferic Nervous System	Every cell wrap a single axon segment forming the neurolemmas (the external layers facilitating axon regeneration)

## Data Availability

No new data were created or analyzed in this study. Data sharing is not applicable to this article.
